# Central venous pressure measurement is associated with improved outcomes in critically ill elderly patients: A propensity score matched cohort study based on the MIMIC-III database

**DOI:** 10.1097/MD.0000000000048079

**Published:** 2026-03-20

**Authors:** Jun Wang, Hong-Chun Ding, Yang-Ru-Rou Liu, Shun-Bi Liu, Li Jiang, Feng Gao

**Affiliations:** a ICU, Department of Cardiovascular Surgery, People’s Liberation Army The General Hospital of Western Theater Command, Chengdu, Sichuan, China.

**Keywords:** 28-day mortality, central venous pressure, critically ill, elderly, lactate

## Abstract

To examine the association of early central venous pressure (ECVP) measurement with 28-day mortality in critically ill elderly patients. We used the Medical Information Mart for Intensive Care database to identify critically ill elderly patients. Multivariable logistic regression was used to control confounding effects and characterize the association between ECVP measurement and 28-day mortality. Propensity score matching and propensity score-based inverse probability of treatment weighting were employed to assess the robustness of our findings. 21,781 patients were included in our study, of which 2860 underwent central venous pressure measurement within 24 hours of intensive care unit admission (ECVP group). ECVP measurement was associated with lower 28-day mortality (odds ratio = 0.73, 95% Confidence Interval 0.634–0.84, *P* < .001). The ECVP group’s in-hospital mortality and 1-year mortality were also significantly lower than those of the non-ECVP group. The ECVP patients had significantly shorter duration of intensive care unit stay and hospital stay. The ECVP patients received more intravenous fluid. The duration of mechanical ventilation and vasopressor use among ECVP patients was also shorter. The ECVP group lactate increase was significantly lower than the non-ECVP group. The mediating effect of lactate change was significant (*P* = .014 for the average causal mediation effect). ECVP measurement is associated with a decreased risk of 28-day mortality, and serum lactate change may proportionally mediate this beneficial effect.

## 1. Introduction

As elderly patients admitted to the intensive care unit (ICU) are susceptible to a variety of morbidities and deaths, they pose a major challenge in intensive caremedicine. Haas et al observed higher hospital mortality and medical expenses in older patients correspondingly increased as the age group of patients increased.^[1,2]^

Central venous pressure (CVP) is an important component of hemodynamic monitoring and essential in managing critically ill elderly patients. Fluid therapy may increase cardiac output and improve perfusion; however, inappropriate fluid administration may increase hydrostatic pressure, causing or worsening edema. CVP has been included in early goal-directed therapies to predict fluid status and responsiveness.^[3]^ Compared to those with a high CVP value, patients with a low CVP value are more likely to benefit from fluid administration. Matthieu Biais et al observed a positive response to fluid in patients with CVP values < 6 mm Hg, and a negative response to fluid in patients with CVP values > 15 mm Hg.^[4]^ Legrand et al demonstrated in their study that patients with high CVP values within the first 24 hours after admission were more likely to develop acute kidney injury (AKI).^[5]^ Smyrniotis et al found that limiting CVP at a lower level was associated with improved postoperative outcomes.^[6]^ Additionally, the CVP waveform carries important information, such as compliance of the right ventricle and chest wall, pericardial effusion, and inspiratory effort.^[7]^ Venn et al demonstrated in their prospective randomized controlled trial that CVP measurement combined with esophageal doppler ultrasonography shortened hospital stays in patients who had received proximal femoral fracture repair.^[8]^ Lin et al found improved clinical outcomes in critically ill septic patients treated with a goal-directed protocol, which included CVP, mean arterial pressure, and urine output.^[9]^ However, the validity of CVP management has been challenged in some recent studies and guidelines,^[10–12]^ and the association between early central venous pressure (ECVP) measurement and outcomes of critically ill elderly patients has not been given proper attention. Additionally, CVP can guide various interventions, such as fluid therapy, to improve tissue perfusion and oxygen availability.^[13]^ Hypoperfusion may lead to lactate elevation, hyperlactatemia is associated with long-term mortality,^[14]^ and lactate reduction is correlated with reduced mortality.^[15]^ However, lactate levels in critically ill elderly patients may not be necessarily lower after short-term treatment, such as 3 days. Therefore, we hypothesized that serum lactate change may mediate the effect of ECVP measurement on outcomes.

In this study, we aimed to investigate the association between ECVP measurement and 28-day mortality in critically ill elderly patients.

## 2. Methods

### 2.1. Study design

This was a retrospective, longitudinal, single-center cohort study of elderly patients from the Medical Information Mart for Intensive Care (MIMIC-III) database, which is a large database containing comprehensive clinical data from thousands of admissions to ICUs at the Beth Israel Deaconess Medical Center.^[16]^ The MIMIC-III database was approved by the Institutional Review Boards of the Massachusetts Institute of Technology and Beth Israel Deaconess Medical Center, which waived Institutional Review Boards approval from our institution. One author (Jun Wang) obtained access to the database and was responsible for data extraction (certification number 10145962). We used structured query language to extract data. This study is reported by the strengthening the reporting of observational studies in epidemiology statement.^[17]^

### 2.2. Participant selection

Patients over 65 years old were eligible for inclusion. If a patient was admitted to the ICU multiple times, only the first ICU admission was included. If a patient stayed in the ICU for < 24 hours, whether because of transmission to other wards or death, he/she was exclude from this study. We assigned the included patients to 2 groups: those with initial CVP measurements within 24 hours of ICU admission were designated as the ECVP group, and the remaining included patients formed the non-ECVP group.

### 2.3. Outcomes

The primary outcome was 28-day mortality after ICU admission. Secondary outcomes included in-hospital and 1-year mortality; the incidence of AKI (diagnosed according to the Kidney Disease Improving Global Outcomes criteria) during the first 48 hours and 7 days after ICU admission; length of hospital and ICU stay; the number of vasopressor-free, and ventilator-free days within 28 days after ICU admission; the volumes (ml) of intravenous fluid (IVF) in the first 3 days after ICU admission; and serum lactate change (the maximum lactate level on day 1 minus that on day 3).

### 2.4. Covariates

To reduce confounding bias, several covariates were collected according to the reported confounding factors and the study entry survey, including demographic information (age, weight, gender, admission type, ethnicity, and care unit), vital signs (heart rate, respiratory rate, mean arterial pressure, and body temperature [°C]), severity scores (simplified acute physiology score [SAPS], simplified acute physiology score II [SAPS II], systemic inflammatory response syndrome [SIRS], sequential organ failure assessment [SOFA], and Elixhauser comorbidity score,^[18–22]^) comorbidities (hypertension, diabetes, congestive heart failure, cardiac arrhythmias, malignancy, chronic pulmonary diseases, chronic kidney disease, chronic liver disease, anemias, hypothyroidism, coagulopathy [all comorbidities were collected according to the International Classification of Diseases 9th Edition codes recorded in MIMIC-III]), laboratory results (arterial oxygen partial pressure [PaO^2^], pH, arterial carbon dioxide partial pressure [PaCO^2^], creatinine, blood glucose, white blood cell count, platelet, hemoglobin, serum lactate, potassium, chlorine, sodium, bicarbonate, international normalized ratio), and interventions during the first 24 hours of ICU admission (vasopressor agents, mechanical ventilation [MV]).

For variables recorded multiple times during the first 24 hours of ICU admission, we selected the values that relate to the greatest severity of the disease.

### 2.5. Statistical methods

Multiple imputation was performed to avoid bias induced by missing data. Given that there were differences in the baseline characteristics between the ECVP group and the non-ECVP group, we employed multivariable logistic regression to control confounding effects and elucidate the association between ECVP measurement and 28-day mortality. Causal mediation analysis (CMA) was used to explore the potential causal mechanism involved with ECVP measurement, serum lactate reduction, and the 28-day mortality. CMA breaks down the total effect of an intervention on the outcome into direct and indirect causal effects (mediated by a mediator). The results include a total effect, an average causal mediation effect (ACME), and an average direct effect.

Sensitivity analysis: Propensity score-based inverse probability of treatment weighting (IPTW) and propensity score matching (PSM) were used to assess the robustness of the results. The patients’ propensity scores for ECVP measurement were estimated by a multivariate logistic regression model of one-to-one nearest neighbor matching with a caliper width of 0.05. A binary regression with an estimand of the average treatment effect was applied to generate inverse probability weights. We conducted logistic regressions in the matched, original, and weighted cohorts. We also conducted subgroup analysis comparing patients whose initial CVP level was above 8 mm Hg or below 8 mm Hg with non-ECVP patients.

Counting data were compared using the Chi-square test and represented as total numbers and percentages. Normally distributed continuous data were compared using a *t* test and represented as means (standard deviations). Non-normally distributed continuous data were compared using a nonparametric test and represented as medians (interquartile ranges [IQR])

All analyses were performed using R (version 4.2.3). Effect sizes were calculated, and a *P* value < .05 was considered statistically significant.

## 3. Results

### 3.1. Selection and clinical characteristics of the study population

**Figure 1.**
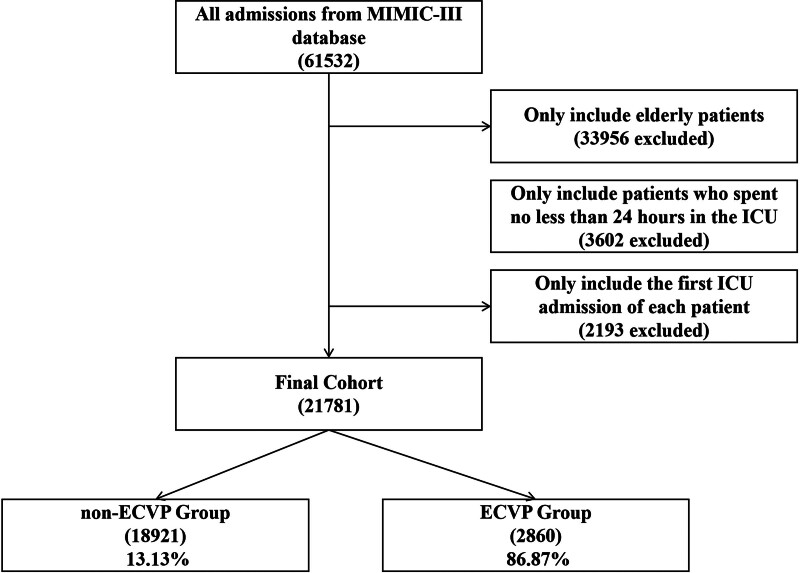
The study flowchart. ECVP = early central venous pressure, ICU = intensive care unit, MIMIC-III = medical information mart for intensive care database.

621,532 admissions were identified in the MIMIC-III database. After excluding patients younger than 65 years old and those who stayed in ICU for < 24 hours, and including only the first ICU admission of each patient, 21,781 patients were finally included in the present study. The selection process is shown in Figure 1.

**Figure 2.**
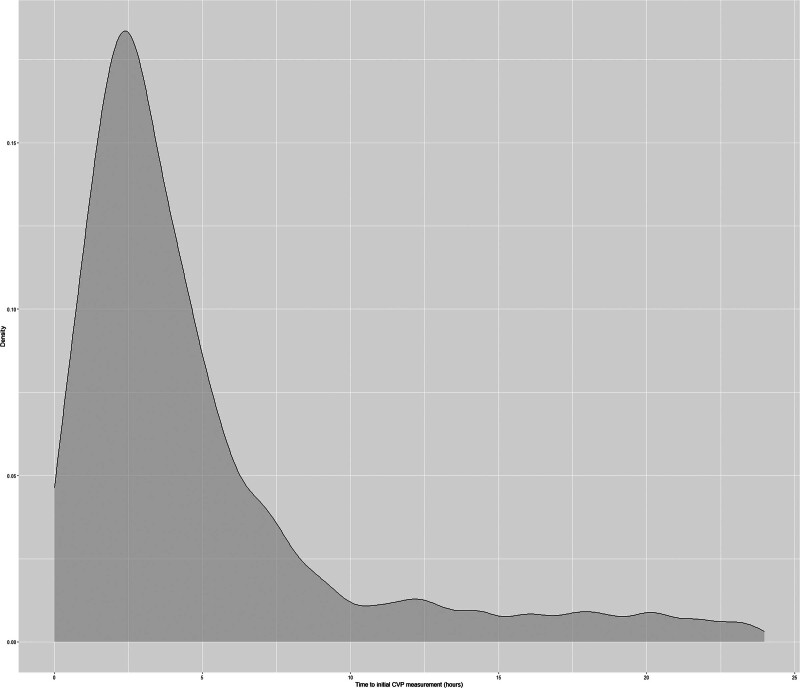
Density of distribution of CVP measurement timing. CVP = central venous pressure.

Of all the included patients, 10,289 (47.2%) were female and 11,492(52.8%) were male, with a median age of 77.47 (IQR, 71.21–83.63). The median initiation time of ECVP measurement was 3.53 (IQR, 1.94–6.91) hours after ICU admission (Fig. 2). The overall 28-day mortality was 17.9%.

In the original cohort, patients in the ECVP group had higher severity scores, including SOFA score (6 [IQR, 4–8] vs 4 [IQR, 2–6]), SIRS criteria (3 [IQR, 2–4] vs 3 [IQR, 2–3]), SAPS II (42 [IQR, 35–53] vs 38 [IQR, 32–47]), and SAPS score (21 [IQR, 19–24] vs 19 [IQR, 16–22]). A larger percentage of ECVP patients received vasopressor agents (78.5% vs 28.5%) and MV (79.2% vs 42.3%) during the first 24 hours of ICU admission. The non-ECVP patients had more emergency admissions (66.8% vs 85.6%) and urgent admissions (1.4% vs 2.7%). After PSM, those between-group differences were minimized. The characteristics of the original cohort and PSM cohort are summarized in Table 1.

**Table 1:** Baseline characteristics of the original cohort and the matched cohort.

Covariates	Original cohort				Matched cohort				Missing data (%)
	Non-ECVP group	ECVP group	*P*	SMD	Non-ECVP group	ECVP group	*P*	SMD	
N	18,921	2860			2616	2616			NA
Age (yr)	77.68 (71.39–83.85)	75.93 (69.99–82.15)	<.001	0.18	76.28 (70.61–82)	76.03 (70.05–82.3)	.646	0.008	0
Weight (kg)	74 (62.4–86.8)	78.8 (67–90.7)	<.001	0.189	77.2 (65.8–90)	78.4 (67–90.5)	.088	0.012	6.3
Male (%)	9832 (52)	1660 (58)	<.001	0.122	1518 (58)	1514 (57.9)	.933	0.003	0
Admission type (%)			<.001	0.507			.949	0.009	0
Elective	2210 (11.7)	910 (31.8)			769 (29.4)	772 (29.5)			
Emergency	16,195 (85.6)	1910 (66.8)			1805 (69)	1805 (69)			
Urgent	516 (2.7)	40 (1.4)			42 (1.6)	39 (1.5)			
Ethnicity (%)			<.001	0.131			.832	0.034	0
Asian	422 (2.2)	69 (2.4)			67 (2.6)	58 (2.2)			
Black	1393 (7.4)	141 (4.9)			122 (4.7)	133 (5.1)			
Hispanic	346 (1.8)	65 (2.3)			58 (2.2)	56 (2.1)			
White	14,165 (74.9)	2259 (79)			2066 (79)	2053 (78.5)			
Other/Unknown	2595 (13.7)	326 (11.4)			303 (11.6)	316 (12.1)			
Care unit (%)			<.001	0.876			.67	0.042	0
CCU	3520 (18.6)	179 (6.3)			171 (6.5)	178 (6.8)			
CSRU	3082 (16.3)	1532 (53.6)			1303 (49.8)	1315 (50.3)			
MICU	7447 (39.4)	749 (26.2)			716 (27.4)	729 (27.9)			
SICU	3003 (15.9)	237 (8.3)			266 (10.2)	234 (8.9)			
TSICU	1869 (9.9)	163 (5.7)			160 (6.1)	160 (6.1)			
Vital signs									
HR (bpm)	99 (87–114)	100 (89–113)	<.001	0.074	100 (90–115)	100 (90–114)	.398	0.03	0.8
MAP (mm Hg)	56 (50–63)	54 (48–59)	<.001	0.333	53 (47–58)	54 (48–59)	.007	0.019	0.8
RR (bpm)	26 (23–31)	26 (23–30)	.003	0.089	26 (23–30)	26 (23–30)	.557	0.037	0.8
Temperature (°C)	36.06 (35.6–36.44)	35.9 (35.44–36.39)	<.001	0.201	35.89 (35.4–36.33)	35.9 (35.44–36.39)	.065	0.028	2.9
SAPS	19 (16–22)	21 (19–24)	<.001	0.471	21 (19–24)	21 (19–24)	.534	0.016	0
SAPS II	38 (32–47)	42 (35–53)	<.001	0.387	42 (34–52)	42 (35–53)	.325	0.012	0
SIRS	3 (2–3)	3 (2–4)	<.001	0.234	3 (2–4)	3 (2–4)	.006	0.048	0
SOFA	4 (2–6)	6 (4–8)	<.001	0.693	6 (4–8)	6 (4–8)	.629	0.005	0
Elixhauser score	5 (0–11)	5 (0–11)	.018	0.014	5 (0–11)	5 (0–12)	.74	0.004	0
Comorbidities									
Hypertension (%)	2936 (15.5)	555 (19.4)	<.001	0.103	455 (17.4)	491 (18.8)	.209	0.036	0
Diabetes (%)	5719 (30.2)	1012 (35.4)	<.001	0.11	898 (34.3)	903 (34.5)	.907	0.004	0
CHF (%)	4622 (24.4)	487 (17)	<.001	0.183	493 (18.8)	476 (18.2)	.569	0.017	0
CA (%)	5352 (28.3)	590 (20.6)	<.001	0.179	592 (22.6)	566 (21.6)	.405	0.024	0
Malignancy (%)	1780 (9.4)	237 (8.3)	.057	0.039	223 (8.5)	224 (8.6)	1	0.001	0
CPD (%)	4355 (23)	679 (23.7)	.392	0.017	607 (23.2)	608 (23.2)	1	0.001	0
CKD(%)	3520 (18.6)	628 (22)	<.001	0.083	529 (20.2)	556 (21.3)	.375	0.025	0
CLD (%)	563 (3)	131 (4.6)	<.001	0.084	125 (4.8)	114 (4.4)	.508	0.02	0
Anemias (%)	4242 (22.4)	808 (28.3)	<.001	0.134	688 (26.3)	701 (26.8)	.707	0.011	0
Hypothyroidism (%)	2462 (13)	432 (15.1)	.003	0.06	385 (14.7)	380 (14.5)	.876	0.005	0
Coagulopathy (%)	1916 (10.1)	507 (17.7)	<.001	0.221	402 (15.4)	433 (16.6)	.257	0.032	0
Laboratory tests									
PO_2_ (mm Hg)	88 (69–122)	90 (73–114)	.222	0.127	87 (71–113)	90 (73–115)	.012	0.001	33.9
pH	7.35 (7.29–7.41)	7.31 (7.26–7.36)	<.001	0.399	7.31 (7.26–7.36)	7.32 (7.26–7.36)	.045	0.026	34
PCO_2_ (mm Hg)	45 (39–52)	47 (42–52)	<.001	0.037	47 (42–53)	47 (42–53)	.75	0.001	34
Creatinine (mg/dL)	1.1 (0.8–1.7)	1.1 (0.8–1.7)	.335	0.032	1.2 (0.9–1.7)	1.1 (0.8–1.7)	.282	<0.001	0.4
Blood glucose(mg/dL)	160 (127–204)	170 (145–203)	<.001	0.052	174 (144–208)	169 (144–204)	.05	0.006	0.4
WBC (×10^9^/L)	12 (8.9–16.2)	14.1 (10.7–18.6)	<.001	0.155	13.3 (10–17.8)	14.1 (10.7–18.52)	<.001	0.013	0.9
Platelet (×10^9^/L)	189 (137–254)	149 (111–203)	<.001	0.376	147 (105.75–212.25)	151 (113–208)	.111	0.01	0.6
Hemoglobin (g/dL)	9.8 (8.6–11.3)	8.6 (7.3–9.8)	<.001	0.653	8.7 (7.5–10)	8.7 (7.5–9.9)	.977	0.003	0.6
Serum lactate (mmol/L)	2.1 (1.4–3.2)	2.6 (1.8–3.7)	<.001	0.2	2.4 (1.6–3.8)	2.6 (1.8–3.7)	<.001	0.008	38.9
Potassium (mmol/L)	4.5 (4.1–5.1)	5 (4.5–5.5)	<.001	0.385	5 (4.4–5.6)	5 (4.4–5.5)	.648	0.014	0.3
Chlorine (mmol/L)	107 (103–110)	110 (107–113)	<.001	0.55	110 (106–113)	110 (107–113)	.61	0.009	0.6
Sodium (mmol/L)	140 (138–143)	140 (138–142)	.06	0.017	140 (138–142)	140 (138–142)	.986	0.008	0.3
Bicarbonate (mmol/L)	23 (20–26)	22 (20–24)	<.001	0.305	22 (19–24)	22 (20–24)	.227	0.019	0.8
INR	1.3 (1.2–1.7)	1.4 (1.3–1.6)	<.001	0.07	1.5 (1.3–1.8)	1.4 (1.3–1.6)	<.001	0.025	8.4
Interventions (1st 24 h)									
Vasopressor (%)	5401 (28.5)	2246 (78.5)	<.001	1.158	2033 (77.7)	2003 (76.6)	.34	0.027	0
MV (%)	8010 (42.3)	2266 (79.2)	<.001	0.816	2012 (76.9)	2024 (77.4)	.717	0.011	0

Data are presented as medians (interquartile range) or means ± standard deviations or n (%).

CA = cardiac arrhythmia, CCU = coronary care unit, CHF = congestive heart failure, CKD = chronic kidney disease, CLD = chronic liver disease, CPD = chronic pulmonary disease, CSRU = cardiac surgery unit, CVP = central venous pressure, ECVP = early central venous pressure, HR = heart rate, INR = international normalized ratio, MAP = mean arterial pressure, MICU = medical intensive care, MV = mechanical ventilation, N = number of patients, PO2 = partial pressure of oxygen; PCO2, partial pressure of carbon dioxide RR = respiratory rate, SAPS II = Simplified Acute Physiology Score II, SAPS = Simplified Acute Physiology Score, SICU = surgical intensive care unit, SIRS = Systemic inflammatory response syndrome criteria, SMD = standardized mean differences, SOFA = Sequential Organ Failure Assessment, TSICU = trauma surgical intensive care unit, WBC = white blood cell, yr = year.

### 3.2. Primary outcome and sensitivity analysis

**Figure 3.**
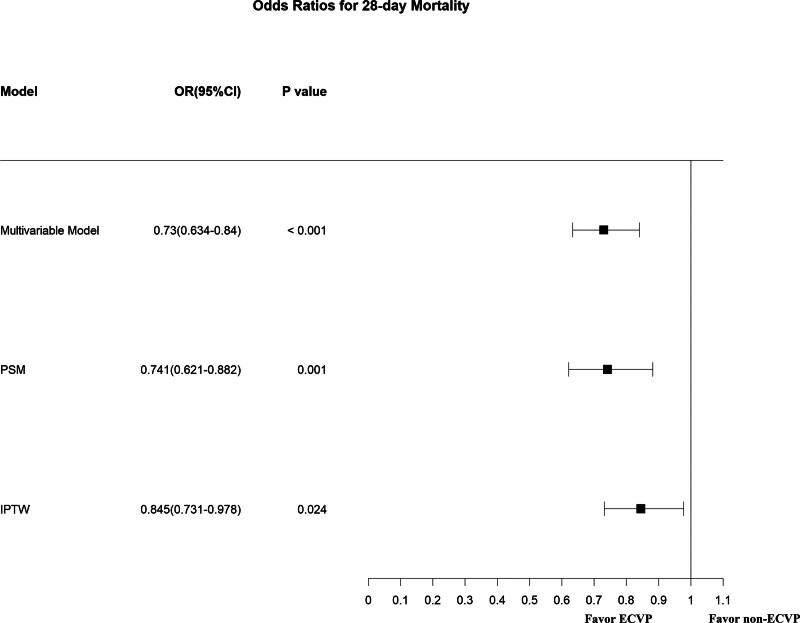
Association between CVP measurement and 28-day mortality. CI = confidence interval, CVP = central venous pressure, ECVP = early central venous pressure, IPTW = inverse probability of treatment weighting, OR = odds ratio, PSM = propensity score matching.

The logistic regression analysis demonstrated a significant beneficial effect of ECVP measurement in terms of the 28-day mortality (odds ratio = 0.73, 95% Confidence Interval [CI 0.634–0.84, *P* < .001). The association remained robust after PSM (odds ratio = 0.741, 95% CI 0.621–0.882, *P* = .001) and IPTW (odds ratio = 0.845, 95% CI 0.731–0.978, *P* = .024) (Fig. 3). After PSM, the 28-day mortality for the ECVP group was significantly lower (15.5% vs 17.9%, *P* = .026) (Table 2).

**Table 2:** Clinical outcomes analysis with propensity score-matched cohorts.

	Non-ECVP group	ECVP group	*P*	SMD	Missing data (%)
28-day mortality (%)	467 (17.9)	406 (15.5)	.026	0.063	0
In-hospital mortality (%)	426 (16.3)	366 (14)	.023	0.064	0
1-year mortality (%)	896 (34.3)	731 (27.9)	<.001	0.137	0
48-hour AKI (%)	2538 (97)	2604 (99.5)	<.001	0.195	0
7-day AKI (%)	2568 (98.2)	2611 (99.8)	<.001	0.165	0
ICU stay (days)	3.1 (1.91–6.06)	2.96 (1.7–5.31)	.001	0.108	0
Hospital stay (days)	8.89 (5.97–14.27)	8.11 (5.44–12.89)	<.001	0.104	0
Vasopressor-free days in 28 days	27.4 (26.36–27.96)	27.56 (26.54–27.98)	.001	0.095	0
Ventilation-free days in 28 days	25.8 ± 4.87	26.06 ± 4.09	.032	0.059	0
IVF (ml)					
Total amount (first 3 days)	3000 (1500–5125)	3900 (2197.12–5959.25)	<.001	0.129	26.6
First day	2073 (1000–3750)	3000 (1500–4500)	<.001	0.253	37.6
Second day	500 (360–1000)	500 (350–1000)	<.001	0.073	68.1
Third day	375 (279–750)	400 (285.75–750)	.004	0.056	82.4
Lactate increase (mmol/L)	0.19 ± 0.9	0.13 ± 0.69	.011	0.071	38.9

Data are presented as medians (interquartile range) or means ± standard deviations or n (%).

AKI = acute kidney injury, CVP = central venous pressure, ECVP = early central venous pressure, ICU = intensive care unit, IVF = intravenous fluid, SMD = standardized mean differences.

### 3.3. Secondary outcomes with propensity score matched cohorts

1. Survival data: Compared with the ECVP patients, a larger percentage of the non-ECVP patients died in hospital (14% vs 16.3%, *P* = .023) and within 1 year (27.9% vs 34.3%, *P* < .001).
2. Treatment efficiency indicators: The ECVP patients had significantly shorter duration of ICU stay (2.96 [IQR, 1.7–5.31] days vs 3.1 [IQR, 1.91–6.06] days, *P* = .001) and hospital stay (8.11 [IQR, 5.4412.89] days vs 8.89 [IQR, 5.97–14.27] days, *P* < .001). The duration of MV (ventilation-free days on day 28 of 26.06 ± 4.09 vs 25.8 ± 4.87, *P* = .032), vasopressor use (vasopressor-free days on day 28 of 27.56 [IQR, 26.54–27.98] vs 27.4 [IQR, 26.36–27.96], *P* = .001) among ECVP patients were also shorter. The lactate increase in the ECVP group was significantly lower compared to the non-ECVP group (0.13 ± 0.69 vs 0.19 ± 0.9).
3. Fluid Management and Metabolism: The amount of IVF was significantly more for the ECVP patients (total, 3900 [IQR, 2197.12–5959.25] ml vs 3000 [IQR, 15005125] ml, *P* < .001; first day, 3000 [IQR, 1500–4500] ml vs 2073 [IQR, 1000–3750] ml, *P* < .001; second day, 500 [IQR, 3501000] ml vs 500 [IQR, 3601000] ml, *P* < .001; third day, 400 [IQR, 285.75–750] ml vs 375 [IQR, 279750] ml, *P* = .004). (Table 2)

### 3.4. Causal mediation analysis

**Figure 4.**
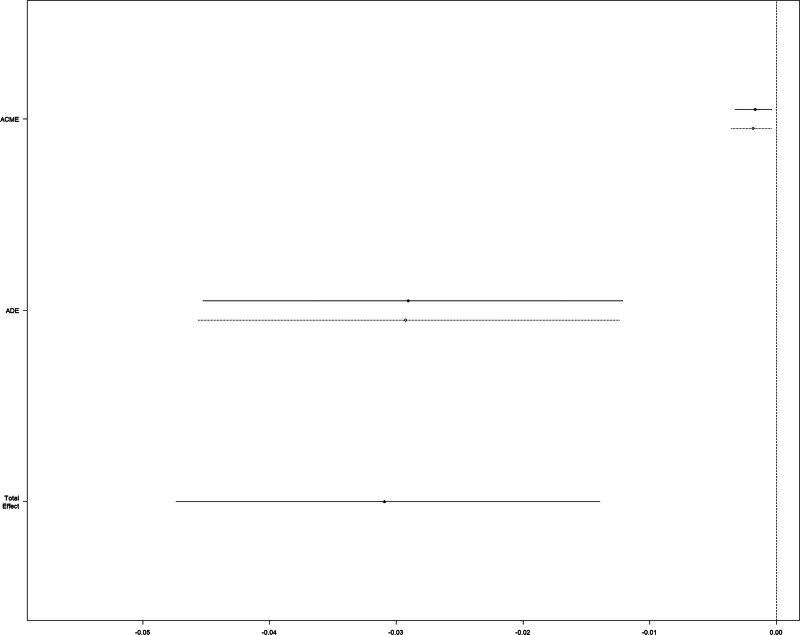
Causal mediation analysis for lactate increase. The solid line represents the ECVP group, and the dashed line represents the non-ECVP group. ACME = average causal mediation effect, ADE = average direct effect, ECVP = early central venous pressure.

The CMA suggested that the increase of serum lactate mediated 5.689% (95% CI 1.16–15%; *P* = .014) of the beneficial effect of CVP (*P* = .014 for ACME) in terms of 28-day mortality (Fig. 4).

### 3.5. Subgroup analyses

**Figure 5.**
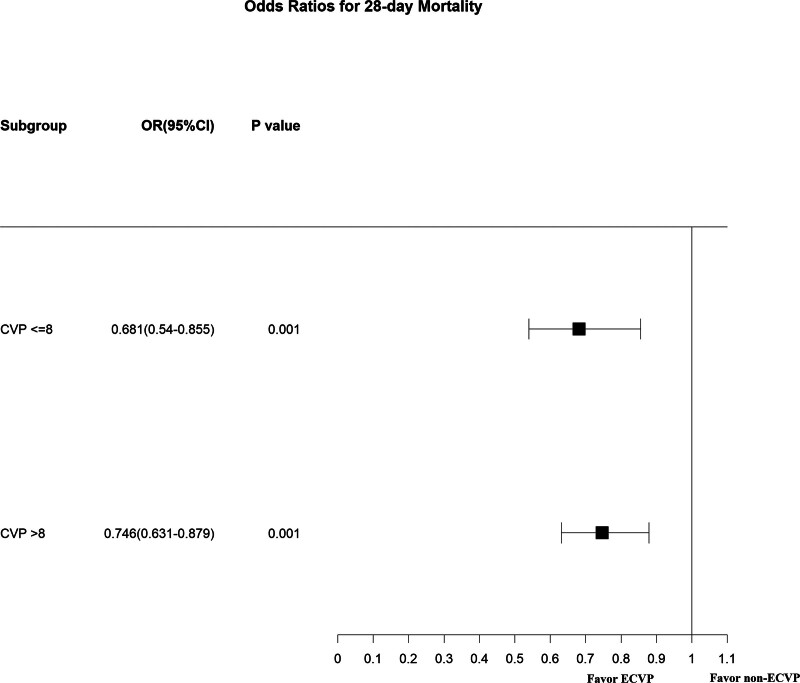
Association between early CVP measurement and 28-day mortality in subgroups. CVP = central venous pressure, ECVP = early central venous pressure, OR = odds ratio.

**Figure 6.**
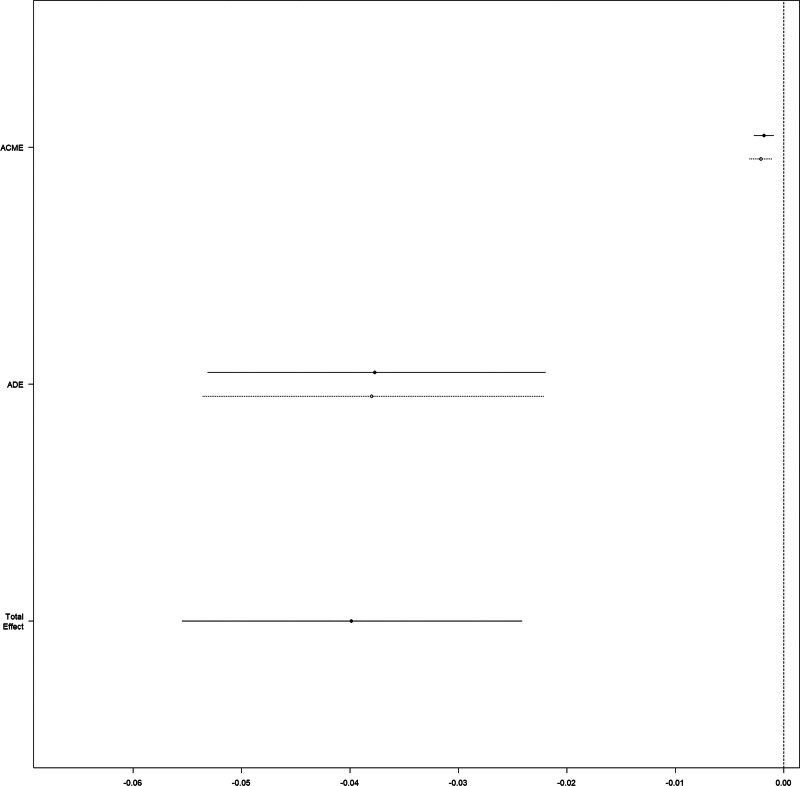
Causal mediation analysis for lactate increase in the subgroup1 (CVP > 8mm Hg). ACME = average causal mediation effect, ADE = average direct effect, CVP = central venous pressure

**Figure 7.**
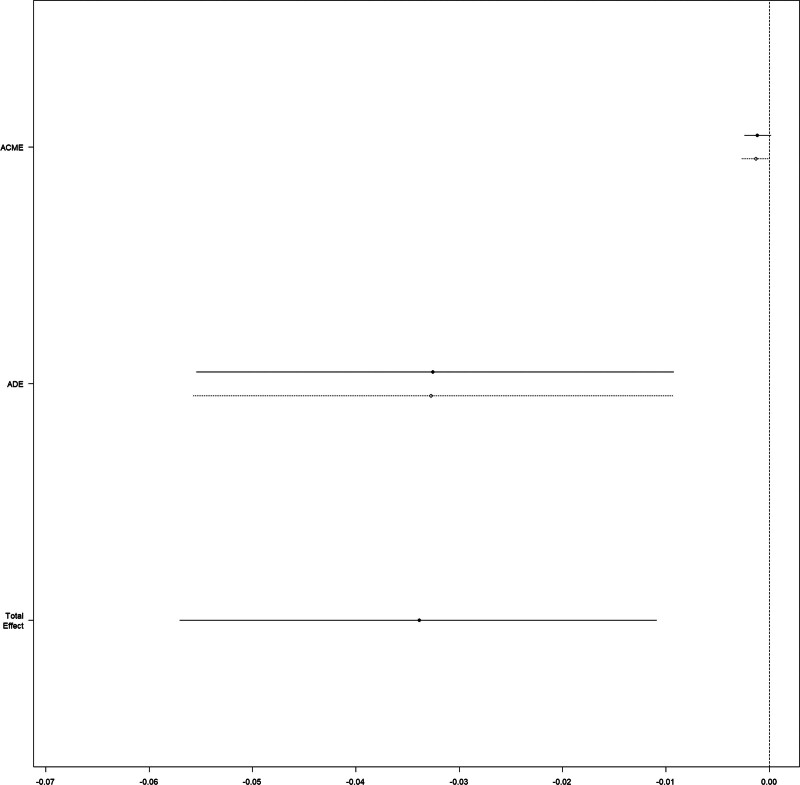
Causal mediation analysis for lactate increase in the subgroup2 (CVP < 8mm Hg). ACME = average causal mediation effect, ADE = average direct effect, CVP = central venous pressure

We contrasted patients with a CVP level below or over 8 mm Hg against the non-ECVP patients. The beneficial effect of ECVP measurement (Fig. 5) remained associated with decreased 28-day mortality. The mediation effect of serum lactate change only remained significant in patients with a CVP level over 8 mm Hg (*P* < .001 for the ACME) (Fig. 6), but not in patients with a CVP level below 8 mm Hg (Fig. 7).

## 4. Discussion

Our results demonstrated that ECVP measurement was associated with lower 28-day mortality, in-hospital mortality, and 1-year mortality than non-ECVP measurement. Serum lactate change had a significant mediating effect on ECVP measurement in terms of 28-day mortality.

The overall 28-day mortality of the whole cohort was 17.9%, which is consistent with the data of previous studies.^[23,24]^

CVP has been utilized for several decades to guide fluid therapy,^[25]^ but the theory of the physiological mechanism of CVP and how it influences clinicians’ decisions has been updated in recent years. However, the role of ECVP measurement on the outcomes of critically ill elderly patients has never been questioned. With the rapid development of new technologies and their application to clinical practice, such as critical care ultrasound and pulse index contour continuous cardiac output, some old techniques are losing advantage and even being abandoned, such as CVP. Nonetheless, because of the uneven development of medical care in different countries and regions, many patients in underdeveloped areas do not have access to advanced monitoring approaches. Therefore, reassessment of the value of some old techniques is important, which has been made easier with the development of electronic medical records producing real-world data.^[26]^

In the present study, the lactate level as well as severity scores, including SOFA score, SIRS criteria, SAPS II score, and SAPS score, were significantly higher in the ECVP group than those in the non-ECVP group. In addition, patients in the ECVP group were more likely to be treated with MV and vasopressors in the first 24 hours. The above information indicates that patients in the ECVP group might start with worse conditions. However, we found significantly better prognostic outcomes in patients in the ECVP group than in those in the non-ECVP group after adjusting for confounding factors, and this benefit persisted regardless of the initial CVP level.

First, logistic regression, confirmed by PSM and IPTW, showed that ECVP measurement was associated with a significantly reduced 28-day mortality. In addition, the 28-day mortality, in-hospital mortality, and 1-year mortality in the ECVP group were significantly lower than those in the non-ECVP group after adjusting for confounding factors. Moreover, patients in the non-ECVP group developed more AKI during the first 48 hours and 7 days compared to those in the ECVP group, and the ECVP patients weaned off MV and vasopressors faster than patients in the non-ECVP group, indicating that ECVP measurement may have a beneficial effect on organ protection and circulation. Additionally, patients in the ECVP group received more IVF, and patients in the non-ECVP group had a greater increase in serum lactate, suggesting that ECVP measurement may lead to sufficient fluid resuscitation and better perfusion.

ECVP measurement may influence mortality by affecting other variables. However, exploring such effects in a retrospective cohort study was difficult. Therefore, we used CMA to address this limitation. We observed a greater increase in serum lactate in the non-ECVP group than in the ECVP group. Hence we hypothesized that some interventions related to ECVP measurement could minimize the increase in serum lactate in critically ill elderly patients. We used ECVP measurement as the treatment and the increase in serum lactate from day 1 to day 3 as a mediator variable in the CMA analysis and found that serum lactate change had a significant mediating effect on ECVP measurement in terms of 28-day mortality. This may be explained by the fact that ECVP patients received more IVF because insufficient fluid resuscitation could lead to a greater increase in serum lactate.^[27]^

CVP level is also an important variable in the treatment of elderly patients in the ICU. In their systematic review, Eskesen et al found that a lower CVP level may better predict fluid responsiveness compared to a higher CVP level.^[11]^ Immediate fluid resuscitation was recommended in patients suffering shock associated with parameters of low levels of preload including CVP.^[12]^ A high CVP level should be utilized neither to predict fluid responsiveness nor to guide fluid resuscitation, but it might serve as a safety endpoint.^[28–30]^ Hence, we conducted subgroup analyses of patients with lower CVP levels ( ≤ 8mmHg) and higher ones ( ≥ 8mmHg), which showed that the relationship between ECVP measurement and 28-day mortality remains robust for patients with different initial CVP levels. Therefore, according to our results, which demonstrated a beneficial effect of ECVP measurement in elderly patients regardless of the initial CVP level in real-world clinical practice, ECVP measurement should never be abandoned but should be used in appropriate ways.

Our findings indicate that ECVP measurement provides information that may aid in the management of critically ill elderly patients. We acknowledge that careful and rigorous statistical analysis is needed in this kind of observational study to ensure that the results are valid and useful for clinical decision-making. We believe that we have achieved this in our study and plan to conduct further analyses in the future to reduce ambiguity and improve decision-making in the complex ICU environment. Nonetheless, our study still carries some limitations. First, the data of the MIMIC-III database were from patients admitted between 2001 and 2012, so our results may not reflect current practices. Second, some important data were not recorded, such as interventions before and after ECVP measurement, so further exploration was not possible. Third, absolute contraindications (e.g., untreated coagulopathy) may had existed among patients without CVP measurement, the database’s lack of detailed clinical notes prevented targeted exclusion, introducing potential confounding. Therefore, we employed multivariable regression models, PSM and IPTW to balance baseline characteristics. Subgroup analyses further focused on patients with CVP measurements, excluding those without CVP data to minimize bias from unmeasured contraindications. Fourth, the relationship between ECVP measurement and mortality was not thoroughly investigated, and because of the complicated clinical practice characteristics, ECVP measurement may have nothing to do with lactate change.^[31]^ Those problems should be addressed in future studies. Fourth, while our study provides evidence within a defined elderly ICU population, the generalizability to other groups, such as trauma patients, remains to be established. Future prospective research in these broader populations is necessary to confirm the utility of CVP monitoring and to further refine its clinical application for the benefit of diverse critically ill patients. In conclusion, ECVP measurement was associated with decreased 28-day mortality in critically ill elderly patients regardless of the initial CVP level. The serum lactate increase may have mediated this effect.

## Author contributions

**Conceptualization**: Feng Gao.

**Data curation:** Jun Wang, Hong-Chun Ding, Yang-Ru-Rou Liu.

**Formal analysis:** Hong-Chun Ding, Yang-Ru-Rou Liu, Shun-Bi Liu, Li Jiang.

**Writing – original draft:** Jun Wan.

**Writing – review & editing:** Hong-Chun Ding, Yang-Ru-Rou Liu, Shun-Bi Liu, Li Jiang, Feng Gao.
